# Impact of discontinuity in health insurance on resource utilization

**DOI:** 10.1186/1472-6963-10-195

**Published:** 2010-07-06

**Authors:** Ritesh Banerjee, Jeanette Y Ziegenfuss, Nilay D Shah

**Affiliations:** 1 Division of Health Care Policy & Research, Mayo Clinic, Rochester Minnesota USA; 2 Knowledge and Encounter Research Unit, Mayo Clinic, Rochester Minnesota USA; 3 Formerly of the Division of Health Care Policy & Research, Mayo Clinic, Rochester Minnesota USA; 4 Analysis Group, Inc., 111 Huntington Avenue, Tenth Floor, Boston, MA 02199, USA

## Abstract

**Background:**

This study sought to describe the incidence of transitions into and out of Medicaid, characterize the populations that transition and determine if health insurance instability is associated with changes in healthcare utilization.

**Methods:**

2000-2004 Medical Expenditure Panel Survey (MEPS) was used to identify adults enrolled in Medicaid at any time during the survey period (n = 6,247). We estimate both static and dynamic panel data models to examine the effect of health insurance instability on health care resource utilization.

**Results:**

We find that, after controlling for observed factors like employment and health status, and after specifying a dynamic model that attempts to capture time-dependent unobserved effects, individuals who have multiple transitions into and out of Medicaid have higher emergency room utilization, more office visits, more hospitalizations, and refill their prescriptions less often.

**Conclusions:**

Individuals with more than one transition in health insurance status over the study period were likely to have higher health care utilization than individuals with one or fewer transitions. If these effects are causal, in addition to individual benefits, there are potentially large benefits for Medicaid programs from reducing avoidable insurance instability. These results suggest the importance of including provisions to facilitate continuous enrollment in public programs as the United States pursues health reform.

## Background

Transitions into and out of health insurance coverage can bring a number of deleterious consequences, both for the health care of the enrollee and for the accountability of providers and insurers (i.e. pay-for-performance programs). Transitions between coverage status can be particularly problematic for individuals with public program coverage in the United States. Adults qualify for Medicaid based on a number of characteristics including income, having children, being pregnant, and/or being disabled. Both eligibility requirements and need to verify eligibility status varies from state to state. Disruptions in Medicaid coverage are common,[1] even among individuals with chronic diseases[2]. Individuals can transition from Medicaid either due to a change in the criteria that made them eligible (i.e., income as a percent of poverty, being pregnant, or having a child that ages out of the program) or because they passively do not enroll from one period the next.

With respect to health care delivery, gaining and then losing insurance coverage may translate into moving between different providers and may lead to discontinuous delivery of care. This would also make it difficult to establish a usual source of care and form a relationship with a medical provider that can lead to an enhanced patient-provider relationship. Additionally, by changing insurance status and potentially also providers, there is a greater likelihood of increased diagnostic testing and imaging. With respect to accountability, a given insurer may not have enough continuity to ensure that its enrollees get the appropriate preventive care.

Individuals with health insurance are known to receive more timely and appropriate care than their counterparts without coverage[3]. While the role of intermittent coverage is less well understood, there is mounting evidence that this impacts receipt of appropriate services. A study of the termination of Medicaid benefits in California demonstrated a significant drop in access to and utilization of health services as well as worse health status after Medicaid eligibility was discontinued[4,5]. Other studies have generally found that compared with people who have health insurance, those without coverage have lower rates of preventive service use,[6-8] lack a usual source of care,[9]and report no physician visits over a period of time[9].

Little is known about insurance instability as specifically measured by the number of disruptions in coverage and the effect of those disruptions on health care delivery. Given previous findings, it is reasonable to presume that when most individuals lose their Medicaid coverage without adequate replacement, they stop consuming health services or change their utilization patterns. In such situations, the health status of some individuals, especially those with chronic conditions, may deteriorate to the point where acute care is needed. One of the only studies to look at the effect of interruptions in Medicaid coverage found that individuals with a diagnosis of schizophrenia who experienced interruptions in their coverage used significantly more inpatient psychiatric services than individuals who had continuous Medicaid coverage[10]. That study compared utilization by individuals with interruptions in coverage and those with continuous coverage but did not distinguish whether utilization of inpatient services occurred before or after interruptions in coverage. Another study reported that insurance transitions, which include a period of no coverage, were associated with postponed care and prescriptions[11]. Other analysis has found that children who experienced a disruption in coverage had lower vaccination coverage as compared with those with continuous insurance[12,13]. Much of this literature evaluating the effects of disruption in insurance has used cross-sectional analyses, and thus have not accounted for changes over time. It is important to consider these changes because the disruption may be due to changes in employment, health status or other factors that may affect insurance transitions.

There is little known about health insurance "movers" - people whose insurance status changes multiple times in a short interval. Are such individuals likely to use more or less health services? Does this answer depend on the type of health service in question? In this paper we use longitudinal national survey data to estimate the impact of transitions into and out of Medicaid, on resource utilization. Additionally, we use dynamic models to estimate our results, as opposed to static models that have been used in previous studies[14,15].

## Methods

### Data

Data for our analysis come from the 2000-2004 Medical Expenditure Panel Survey (MEPS), a nationally representative sample of the non-institutionalized, civilian U.S. population. Individuals are interviewed five times over a 2-year period. We limit our analysis to individuals who report Medicaid enrollment in at least one round of the survey regardless of other types of coverage they may also report. We further limit our analysis to adults between 18 and 64 years of age due to the different eligibility criteria for adults compared to children and the elderly. We retained only individuals who had data for both years of the survey, leaving 6,247 unique individuals. MEPS interviewers are trained to seek out changes in life circumstances from the previous interview resulting in a rich source of time-varying information about the respondents on factors like employment and health status. We attempted to take full advantage of this richness by conducting a longitudinal analysis that used data from all five rounds.

Our primary outcomes of interest are the number of emergency room visits, outpatient office visits, hospitalizations and prescription drug fills, all of which are measured in each round. Our two main independent variables are indicators for one and multiple insurance transitions into or out of Medicaid over the 2-year period. Because the sample was defined based on Medicaid enrollment at some point during the survey period, individuals with no transitions were always enrolled in Medicaid, those with one transition either entered or left Medicaid once, and those with multiple transitions were enrolled in Medicaid for varying timeframes over the survey period. In our baseline model we controlled for age, gender, marital status, race (white or not), family size (number of members), whether the person finished high school, employment status (employed or not during the round), self-reported health status (indicating good, very good or excellent perceived health during the round), income (ratio of total household income to the mean for the entire sample), number of chronic conditions as defined in [16] and round fixed effects. In our other specifications, we include the mean of employment status in each round, mean health status in each round, and initial round utilization. We discuss these further below. This research was deemed exempt by the Mayo Clinic IRB because the MEPS data are available publicly.

### Statistical Analysis

As a means-tested public health insurance program, Medicaid was not designed to be a permanent source of insurance for the majority of its beneficiaries[17]. Changes in family composition, work or income circumstances are common reasons for transitions into or out of the program. We first present a description of the prevalence of single and multiple transitions into and out of Medicaid. We then describe sociodemographic characteristics of these distinct populations. In order to control for these socioeconomic differences, we estimate multivariate models. As many of the factors associated with insurance transitions are not observed in our data, our estimation strategy needs to account for these unobservables. Additionally, some of these unobservables may be time-varying, in which case, treating the process as dynamic may better separate the effects of observed explanatory factors from unobserved ones.

We study four types of health care utilization, all of which are discrete and non-negative, justifying the use of count data models to estimate the impact of multiple transitions on utilization. To account for potential time-varying unobserved factors, as well as assess whether health utilization behavior displays persistence, meaning that prior utilization impacts current and future utilization, we estimate both static and dynamic longitudinal models.

Our basic specification specifies the dependent variable *y*_*it *_to have a Poisson distribution with conditional mean(1)

where *z*_*i *_is a vector of independent variables and *c*_*i *_is an unobserved effect, as described in Wooldridge[18] and Nolan[19]. Unfortunately, random effects estimators like these give inconsistent estimates when the unobserved effects are correlated with observed independent variables. Attitudes toward health, how hard people work, etc. are likely to be correlated with health and employment status. Wooldridge [20] suggests parameterizing the individual effects as a way to mitigate these problems and adds a vector of within-individual means for the time-varying independent variables to the specification. This modifies the specification of the conditional mean to be(2)

where  and  is a vector of within-individual means for the time-varying variables, which in our case are health and employment status. The logic underlying this approach is that by adding a function of the independent variables, we absorb some of the correlation that may exist between the unobserved effect and the independent variables. So for example, if employment history is a better indicator of how hardworking an individual is, the mean of employment status over the survey period should, in principle, capture this effect.

Some forms of health care utilization may be persistent (or state-dependent) over time such that past utilization may be positively correlated with current and future utilization. We modify the static specification to account for persistence by estimating a dynamic model in which the conditional mean is(3)

where *h*(.) is some non-decreasing function, and *b*_*i*0 _is a vector of initial conditions. In a dynamic setting like this, the initial condition is not exogenous. For example, imagine two otherwise identical individuals in our data, A and B, who are enrolled in Medicaid for 4 rounds. A enrolls at the beginning of round 1 and exits at the end of round 4, whereas B enrolls at the beginning of round 2 and exits at the end of round 5. Assume that health care costs fall for both A and B once they enroll in Medicaid and stay low for at least one round after they exit. Both individuals would appear as having one transition (A transitions out of, and B transitions into Medicaid), but B's costs would be higher because we observe B in round 1 (during which he is not enrolled). Controlling for initial period utilization is a simple way of adjusting for such an unobserved difference between individuals by acting as a proxy for past utilization. Wooldridge [18] points out that the coefficient on the initial condition is often large and highly significant which is not surprising since it acts as a baseline catchall for unobserved heterogeneity between individuals. See Nolan[19] for a recent application of this method.

All analysis is conducted in StataSE 10 [21].

## Results

Descriptive statistics for our study population are presented in Table 1. Of individuals enrolled in Medicaid at least at some point during a two year time period, over half of the sample (53.2 percent) had no transitions, indicating that they were covered by Medicaid for the entire period. Approximately one-sixth of the respondents (16.7 percent) had multiple transitions into and out of Medicaid over the two years. The remaining individuals either transitioned once to Medicaid or once away from Medicaid over the survey period. There are important differences between the three groups of individuals. Specifically, those with continuous coverage are older, less likely to be married, have less education, smaller family size, less employment, lower self-reported health status, less income, and more chronic conditions compared to those with one or multiple transitions.

**Table 1:** Descriptive Statistics by Number of Transitions

Variable	Transition = 0 Mean or %	Transition = 1 Mean or %	Transition = 1 p-value*	Transition > 1 Mean or %	Transition > 1 p-value**
Age	50.5 (19.852)	34.4 (13.232)	0.0000	38.9 (18.345)	0.0000
Male %	30.9 (.462)	30.1 (.459)	0.1463	29.7 (.457)	0.0731
Married %	28.7 (.452)	41.3 (.492)	0.0000	0.4 (.496)	0.0000
High School %	41.0 (.492)	51.5 (..5)	0.0000	60.0 (.49)	0.0000
Family Size	3.013 (2.025)	3.774 (1.883)	0.0000	3.388 (1.863)	0.0000
White %	63.4 (.482)	72.6 (.446)	0.0000	69.7 (.46)	0.0000
Employed %	17.4 (.379)	48.4 (.5)	0.0000	45.7 (.498)	0.0000
Healthy %	57.2 (.495)	77.9 (.417)	0.0000	77.0 (.421)	0.0000
Income	0.872 (.924)	1.077 (1.226)	0.0000	1.270 (1.44)	0.0000
Chronic count	0.232 (.553)	0.094 (.341)	0.0006	0.119 (.386)	0.0000

**Unique Individuals**	**3322**	**1883**		**1042**	

*T-Test: Transition = 0 vs. 1; **T-Test: Transition = 0 vs. > 1

Standard deviations in parentheses. Note that dummy variables for each round were included in the models but are not reported for purposes of brevity.

Table 2 shows utilization variables for each of the three populations of interest. Those with continuous Medicaid coverage have more office based visits and prescription refills than either of the other populations. Those with continuous coverage also have more ER visits and inpatient discharges than those with one transition.

**Table 2:** Descriptive Statistics by Utilization

Variable	Transition = 0 Mean or %	Transition = 1 Mean or %	Transition = 1 p-value*	Transition > 1 Mean or %	Transition > 1 p-value**
ER Visits	0.142 (.486)	0.123 (.453)	0.0006	0.137 (.494)	0.4817
Inpatient discharges	0.088 (.355)	0.067 (.286)	0.0000	0.083 (.339)	0.2703
Office-based visits	3.334 (7.898)	1.684 (4.908)	0.0000	2.274 (5.769)	0.0000
Prescription refills	9.399 (15.629)	2.667 (7.065)	0.0000	4.052 (9.452)	0.0000

**Unique Individuals**	**3322**	**1883**		**1042**	

*T-Test: Transition = 0 vs. 1; **T-Test: Transition = 0 vs. > 1

Standard deviations in parentheses. Note that dummy variables for each round were included in the models but are not reported for purposes of brevity.

### Emergency Room Visits

We report results from our random effects specifications in Table 3. Column 1 shows estimates of the basic specification (1). Controlling for demographic and socioeconomic characteristics and number of chronic conditions, individuals who rate themselves as having good health visit the emergency room (ER) 50% less often over the survey period than those who do not. Employed individuals visit the ER 20% less often and people with chronic conditions have 25% more visits. Multiple transitions into and out of Medicaid are not significantly associated with the number of ER visits. Interestingly, household income is not significantly associated with ER visits after conditioning on whether an individual is employed or not or perceives themselves to be healthy. Older individuals are slightly less likely to visit the ER and males visit 25% less often than females.

**Table 3:** Emergency Room Visits

	Static Specification (1)	Static Specification (2)	Dynamic Specification (3)
One transition	0.976 (0.051)	0.995 (0.053)	1.031 (0.058)
More than one transition	1.097 (0.066)	1.126* (0.069)	1.175** (0.076)
Age	0.987*** (0.0013)	0.985*** (0.0014)	0.987*** (0.0014)
Male	0.754*** (0.035)	0.745*** (0.035)	0.772*** (0.038)
Married	0.949 (0.044)	0.942 (0.044)	0.936 (0.047)
Graduated high school	0.961 (0.041)	0.972 (0.042)	0.932 (0.042)
White	0.981 (0.044)	0.977 (0.044)	0.908** (0.043)
Family size	0.908*** (0.011)	0.913*** (0.011)	0.912*** (0.012)
Employed	0.816*** (0.037)	0.832*** (0.050)	0.823*** (0.058)
Health (self-reported)	0.495*** (0.018)	0.608*** (0.029)	0.576*** (0.031)
Income	1.012 (0.020)	1.016 (0.020)	1.006 (0.022)
Number of chronic conditions	1.257*** (0.035)	1.230*** (0.035)	1.228*** (0.037)
Average employment status over the observation time frame		1.022 (0.090)	1.057 (0.10)
Average health status (self reported) over the observation time frame		0.587*** (0.045)	0.664*** (0.055)
ER visits in prior time period			0.983 (0.020)
ER visits at baseline			1.668*** (0.078)

Observations	37482	37482	31235
Number of individuals	6247	6247	6247

Note that dummy variables for each round were included in the models but are not reported for purposes of brevity.

Column 2 uses specification (2) where we have added mean employment and health status over the survey period to capture unobserved factors associated with employment and health status. The mean of self-reported health status (over all rounds) has a comparable influence on ER visits as self-reported health status in any given round. This suggests that unobserved or unmeasured health related factors (proxied here by a function of round-specific status) play an important role in determining ER visits. However, average employment status is not important after accounting for round-specific employment status. Multiple transitions to and from Medicaid have a statistically significant impact on the number of ER visits in this specification suggesting again that specification (1) misses unobserved effects.

Column 3 presents the dynamic specification (3). Two points are worth noting. First, there is no evidence of persistence in visits to the ER meaning that a visit to the ER in the previous round is not correlated with a visit in the current round. The second point to note is the large, positive and significant coefficient on the initial condition (initialervis). Wooldridge [18] points out that the size of the coefficient is not unusual in this type of model. It shows unambiguously that unobserved factors, some of which are time-dependent and captured by the initial condition, that is, the number of visits to the ER by an individual in the first round of the survey, play an important role in this model. For example, the number of visits to the ER in round 1 may say something about an individual's health status or proclivity for using ER-based care over and above that captured by self-reported health status. Including the initial condition increases the coefficient on the multiple transition indicator variable and makes it more statistically significant. Thus in a dynamic context, multiple transitions are associated with a 17.5% increase in ER usage.

### Office and Outpatient Visits

Table 4 reports estimates from regressions in which we run our various specifications with number of office and outpatient visits as the dependent variable. The basic specification reveals that, all else equal, older, white, females with at least a high school education are more likely to have office visits. Employed individuals visit the doctor 32% less often and healthy individuals do so 12% less often relative to those who self-identify as being in fair to poor health. The single and multiple transition dummies are both negative and significant. The latter implies that individuals with unstable insurance are 15% less likely to see a doctor in an office or outpatient setting relative to no-transition individuals.

**Table 4:** Outpatient Visits

	Static Specification (1)	Static Specification (2)	Dynamic Specification (3)
One transition	0.664*** (0.025)	0.725*** (0.027)	0.868*** (0.032)
More than one transition	0.843*** (0.037)	0.973 (0.042)	1.103** (0.047)
Age	1.011*** (0.00096)	1.004*** (0.00093)	1.005*** (0.00091)
Male	0.779*** (0.026)	0.740*** (0.024)	0.715*** (0.023)
Married	0.944** (0.022)	0.954** (0.022)	0.954* (0.023)
Graduated high school	1.333*** (0.041)	1.449*** (0.043)	1.284*** (0.038)
White	1.114*** (0.035)	1.176*** (0.035)	1.154*** (0.035)
Family size	0.976*** (0.0064)	0.981*** (0.0063)	0.976*** (0.0065)
Employed	0.682*** (0.011)	0.711*** (0.012)	0.645*** (0.013)
Health (self-reported)	0.881*** (0.0090)	0.933*** (0.0097)	0.943*** (0.011)
Income	1.016*** (0.0059)	1.025*** (0.0060)	1.041*** (0.0065)
Number of chronic conditions	1.066*** (0.0066)	1.060*** (0.0066)	1.058*** (0.0071)
Average employment status over the observation time frame		0.847*** (0.039)	0.969 (0.046)
Average self reported health status over the observation time frame		0.358*** (0.016)	0.416*** (0.018)
Outpatient visits in prior period			0.999*** (0.00025)
Outpatient visits at baseline			1.083*** (0.0041)

Observations	37482	37482	31235
Number of individuals	6247	6247	6247

Note that dummy variables for each round were included in the models but are not reported for purposes of brevity.

Column 2 reveals that average employment and health history are important determinants of office visits. In fact, individuals who on average report good to excellent health are much less likely to not visit the doctor relative to those who report poor to fair health. The size of this coefficient relative to the round-specific self-reported health status again suggests the importance of unobserved health-related factors that play a role in determining office visits.

The dynamic specification in Column 3 reveals a small, negative and significant relationship between office visits in the previous period and those in the current period implying some evidence of cyclicality in doctor visits. Thus individuals who visited the doctor in the last round are marginally less likely to do so in the current round. A key point to note is that when the initial condition is included, the multiple transition indicator becomes positive and significant. In other words, once we condition on the number of visits in the first period, individuals with unstable insurance are more likely to visit the doctor. Factors like an individual's propensity to see the doctor, or perhaps transitory demand (where prior knowledge of an imminent loss of insurance coverage may lead to increased utilization), may be responsible for this finding. From our perspective, this is further evidence that time-dependent unobserved factors are important to account for in this context.

### Hospitalizations

We report hospitalization results in Table 5. The pattern of results is similar to those for ER visits and office visits in that employed and healthy individuals are less likely to have hospitalizations. Multiple transitions are associated with a 24% increased rate of hospitalizations. Controlling for correlated individual effects in column 2 does not change much except increase the coefficient on self-reported health. The dynamic model reveals that unobserved time-dependent factors play a role: the coefficient on multiple transitions rises about 50% relative to column 1, meaning that controlling for the initial number of hospitalizations is important for understanding the effect of insurance instability on the number of hospitalizations.

**Table 5:** Inpatient Hospitalizations

	Static Specification (1)	Static Specification (2)	Dynamic Specification (3)
One transition	1.013 (0.061)	1.020 (0.062)	1.045 (0.069)
More than one transition	1.239*** (0.084)	1.264*** (0.087)	1.366*** (0.10)
Age	0.995*** (0.0015)	0.994*** (0.0015)	0.994*** (0.0016)
Male	0.672*** (0.036)	0.657*** (0.036)	0.717*** (0.041)
Married	1.042 (0.054)	1.039 (0.054)	1.069 (0.060)
Graduated high school	1.110** (0.053)	1.111** (0.054)	1.085 (0.057)
White	1.166*** (0.059)	1.174*** (0.060)	1.215*** (0.067)
Family size	0.978 (0.013)	0.985 (0.014)	0.977 (0.015)
Employed	0.501*** (0.031)	0.482*** (0.042)	0.469*** (0.046)
Healthy (self-reported)	0.483*** (0.022)	0.613*** (0.038)	0.576*** (0.039)
Income	0.991 (0.024)	0.989 (0.025)	0.979 (0.027)
Number of chronic conditions	1.315*** (0.041)	1.285*** (0.041)	1.285*** (0.044)
Average employment status over the observation time frame		1.140 (0.13)	1.147 (0.15)
Average self-reported health status over the observation time frame		0.587*** (0.054)	0.650*** (0.065)
Inpatient hospitalization in prior period			0.960 (0.037)
Inpatient hospitalization at initial period			1.574*** (0.11)

Observations	37482	37482	31235
Number of individuals	6247	6247	6247

Note that dummy variables for each round were included in the models but are not reported for purposes of brevity.

### Prescription Refills

Table 6 reports results where our outcome variable is the number of prescription refills per round. Single and multiple transitions are associated with reduced utilization. The basic specification shows that older, female, and at least high school educated individuals had higher prescription drug utilization, while employed and healthy individuals had lower utilization. Either one or more than one transition was associated with lower prescription drug utilization. The dynamic model reveals some cyclicality across rounds which implies that individuals have fewer fills than they did in the previous round and vice versa. When the initial conditions are included, the magnitude of the transition effect is reduced. This may indicate that there are people with chronic conditions who continue taking medications that they believe are beneficial.

**Table 6:** Prescription Medication Refills

	Static Specification (1)	Static Specification (2)	Dynamic Specification (3)
One transition	0.513*** (0.022)	0.614*** (0.025)	0.704*** (0.029)
More than one transition	0.597*** (0.030)	0.746*** (0.036)	0.809*** (0.038)
Age	1.035*** (0.0011)	1.026*** (0.0010)	1.023*** (0.0010)
Male	0.871*** (0.033)	0.832*** (0.029)	0.830*** (0.029)
Married	1.058*** (0.021)	1.046** (0.020)	1.072*** (0.022)
Graduated high school	1.206*** (0.042)	1.302*** (0.043)	1.203*** (0.039)
White	1.047 (0.035)	1.093*** (0.034)	1.050 (0.033)
Family size	0.936*** (0.0051)	0.941*** (0.0050)	0.943*** (0.0052)
Employed	0.851*** (0.012)	0.893*** (0.013)	0.942*** (0.016)
Healthy (self-reported)	0.870*** (0.0059)	0.895*** (0.0061)	0.905*** (0.0068)
Income	0.982*** (0.0041)	0.987*** (0.0041)	0.985*** (0.0044)
Number of chronic conditions	1.031*** (0.0036)	1.029*** (0.0036)	1.032*** (0.0038)
Average employment status over the observation time frame		0.710*** (0.035)	0.782*** (0.039)
Average self-reported health status over the observation time frame		0.257*** (0.012)	0.335*** (0.016)
Prescription refills in prior period			0.997*** (0.00014)
Prescription refills at initial period			1.073*** (0.0030)

Observations	37482	37482	31235
Number of individuals	6247	6247	6247

Note that dummy variables for each round were included in the models but are not reported for purposes of brevity.

## Discussion & Conclusions

Insurance instability has many perverse outcomes; we find that one of these is a change in utilization patterns. Our estimates indicate that ER use, office visits and hospitalizations rise between 10% and 36% and that use of prescription medications falls by 19% among the unstably insured compared to those with consistent Medicaid coverage. Lack of a continuous source of coverage may cause individuals to overuse expensive sources of care like the ER or put off seeing a doctor until their health deteriorates enough to warrant an inpatient episode. Conversely, patients without coverage are often unable to afford required prescription medications, and this may in turn lead to an inpatient or ER episode. Alternately, those that are stably insured in Medicaid may be distinct from those that are not as indicated by the descriptive characteristics, in that they may be eligible based on a disability or other chronic condition, impacting both their utilization patterns and ease of enrollment relative to those who qualify based on income and assets.

In a study looking at insurance instability among HIV patients, Smith et al.[22] find that changes in health insurance coverage are associated with lower drug utilization. Beyond this study, to our knowledge, there are no other studies evaluating the effect of inconsistent coverage on prescription drug utilization. Our finding of lower prescription drug utilization for individuals with multiple transitions is not surprising and may be affected by a number of issues including the individual not filling prescriptions while transitioning between insurance types/plans, formulary issues that may restrict use, higher cost-sharing among private plans compared to Medicaid, and treatment/prescribing patterns of new providers when a provider change may be necessary. Alternately, as stated above it may be due to underlying population characteristics of those consistently enrolled.

Multiple transitions to and from public programs may have significant implications for health care costs and quality. Administrative costs may be higher for individuals who undergo multiple insurance transitions. Health plans and providers may lose anticipated revenue or incur costs that they may not otherwise incur. Managing and monitoring care and measuring the quality of care also becomes more difficult. Additionally, a recent study suggests that individuals who lose continuity of care tend to feel unsafe if they do not get to see their usual physician[23]. If individuals with continuous coverage do differ on their basis of eligibility, which cannot be determined using MEPS, lessons can be learned about the potential benefits of continuous enrollment evident even in comparison to a population that likely has more chronic health problems. These relationships should be disentangled in further work.

An interesting feature of our findings is the role played by unobserved factors. Adding parameterized versions of time-varying individual variables changes the estimates in certain cases. Moreover, the dynamic specification reveals that initial conditions play an important role in all cases. We believe these are a proxy for time-dependent unobserved effects; for example, certain individuals may be more likely to utilize health care than others and a dynamic model is necessary to capture this factor. These differences in effects are observed when the static specification is changed to a dynamic specification.

As with any observational study, our study has a number of limitations. We were not able to model the directionality of change and the type of insurance the individual transitioned into. We did not have information on why insurance transitions took place which may affect utilization behavior. For example, an individual transitioning due to a job change may have different utilization patterns than one transitioning due to administrative issues or one transitioning due to pregnancy. Another limitation is that we did not have information on the state of residence and thus were not able to control for differing renewal policies across states. Finally, all of the data used is based on self-report. In our analysis we include those who may erroneously report that they have Medicaid (false positives) and omit those that fail to report that they are covered (false negatives). Prior work has shown, however, that the size of these errors is likely small[24].

From a methodological standpoint, Poisson regression has well-known weaknesses, in particular when data are overdispersed. The negative binomial (NB) model is often used as an alternate model in these situations, but as Cameron and Trivedi[25] point out, it does not help when the conditional mean is poorly specified and it is less robust to distributional misspecification than the Poisson model. Moreover, in our case, Wooldridge's[18] approach for dynamic panels has only been developed for Poisson models so we use this specification in our analysis.

If the effects we have found are indeed causal, there are potentially large gains to instituting programs or policies that provide consistent coverage across transitional life events. Increased utilization of health care in circumstances where individuals lose Medicaid coverage as a result of purely administrative reasons is wasteful from both an individual and societal perspective. It can also be argued that similarly wasteful are the transitions that occur among those that are at the border of eligibility and thus regularly transition on and off Medicaid. Moreover, our results suggest that providing better access to prescription medication may be a potentially effective method for maintaining health for those in transition. This in turn may reduce the need for expensive emergent care.

## Competing interests

The authors declare that they have no competing interests.

## Authors' contributions

RB and NS conceived the study, obtained and analyzed the data. RB, JZ, and NS interpreted the data and drafted and edited the manuscript. All authors gave final approval of the manuscript

## Pre-publication history

The pre-publication history for this paper can be accessed here:

http://www.biomedcentral.com/1472-6963/10/195/prepub

## References

[B1] Short PF Graefe DR Schoen C Churn, churn, churn: how instability of health insurance shapes America's uninsured problem Issue Brief (Commonw Fund) 2003 688 1 16 14621666

[B2] Sommers B Loss of health insurance among non-elderly adults in Medicaid Journal of General Internal Medicine 2009 24 1 1 7 10.1007/s11606-008-0792-9 18810555 PMC2607511

[B3] (IOM) Medicine Io Coverage Matters: Insurance and health care Shaping the Future for Health. Washington, DC 2001

[B4] Lurie N Ward NB Shapiro MF Brook RH Termination from Medi-Cal--does it affect health? N Engl J Med 1984 311 7 480 484 6379458 10.1056/nejm198408163110735

[B5] Lurie N Ward NB Shapiro MF Gallego C Vaghaiwalla R Brook RH Termination of Medi-Cal benefits. A follow-up study one year later N Engl J Med 1986 314 19 1266 1268 3517642 10.1056/NEJM198605083141934

[B6] Ayanian JZ Weissman JS Schneider EC Ginsburg JA Zaslavsky AM Unmet health needs of uninsured adults in the United States Jama 2000 284 16 2061 2069 10.1001/jama.284.16.2061 11042754

[B7] Bednarik HL Schone BS Variations in preventive service use among the insured and uninsured: does length of time without coverage matter? Journal of Health Care for the Poor and Underserved 2003 14 403 419 12955919 10.1353/hpu.2010.0529

[B8] Broyles RW Narine L Brandt EN The temporarily and chronically uninsured: does their use of primary care differ? Journal of Health Care for the Poor and Underserved 2002 13 95 111 10.1177/10492080222148647 11836917

[B9] Kasper JD Giovannini TA Hoffman C Gaining and losing health insurance: strengthening the evidence for effects on access to care and health outcomes Med Care Res Rev 2000 57 3 298 318 discussion 319-225 10.1177/107755870005700302 10981187

[B10] Harman JS Manning WG Lurie N Christianson JB Association between interruptions in medicaid coverage and use of inpatient psychiatric services Psychiatr Serv 2003 54 7 999 1005 10.1176/appi.ps.54.7.999 12851437

[B11] Aiken KD Freed GL Davis MM When insurance status is not static: insurance transitions of low-income children and implications for health and health care Ambul Pediatr 2004 4 3 237 243 10.1367/A03-103R.1 15153059

[B12] Blewett LA Davidson G Bramlett MD Rodin H Messonnier ML The Impact of Gaps in Health Insurance Coverage on Immunization Status for Young Children Health Serv Res 2008 in press 10.1111/j.1475-6773.2008.00864.x PMC2653891 18522671

[B13] Smith PJ Stevenson J Chu SY Associations between childhood vaccination coverage, insurance type, and breaks in health insurance coverage Pediatrics 2006 117 6 1972 1978 10.1542/peds.2005-2414 16740838

[B14] Bindman AB Chattopadhyay A Auerback GM Interruptions in Medicaid Coverage and Risk for Hospitalization for Ambulatory Care-Sensitive Conditions Ann Intern Med 2008 149 12 854 860 19075204 10.7326/0003-4819-149-12-200812160-00004

[B15] Federico SG Steiner JF Beaty B Crane L Kempe A Disruptions in insurance coverage: patterns and relationship to health care access, unmet need, and utilization before enrollment in the State Children's Health Insurance Program Pediatrics 2007 120 4 e1009 10.1542/peds.2006-3094 17908722

[B16] Elixhauser A Steiner C Harris DR Coffey RM Comorbidity measures for use with administrative data Med Care 1998 36 1 8 27 10.1097/00005650-199801000-00004 9431328

[B17] Rosenbaum S Medicaid N Engl J Med 2002 346 8 635 640 10.1056/NEJM200202213460825 11856811

[B18] Wooldridge JM Simple solutions to the intial conditions problem in dynamic, nonlinear panel data models with unobserved heterogeneity Journal of Applied Econometrics 2005 20 1 39 54 10.1002/jae.770

[B19] Nolan A A dynamic analysis of GP visiting in Ireland: 1995-2001 Health Econ 2007 16 2 129 143 10.1002/hec.1149 16929486

[B20] Wooldridge JM Simple solutions to the initial conditions problem in dynamic, nonlinear panel data models with unobserved hetero geneity Journal of Applied Econometrics 2005 20 1 39 54 10.1002/jae.770

[B21] StataCorp Stata statistical software: Release 10 2007 College Station, TX: StataCorp LP

[B22] Smith SR Kirking DM The effect of insurance coverage changes on drug utilization in HIV disease J Acquir Immune Defic Syndr 2001 28 2 140 149 10.1097/00126334-200110010-00005 11588507

[B23] Pandhi N Schumacher J Flynn KE Smith M Patients' perceptions of safety if interpersonal continuity of care were to be disrupted Health Expect 2008 11 4 400 408 10.1111/j.1369-7625.2008.00503.x 19076668 PMC2689380

[B24] Call KT Davidson G Davern M Brown ER Kincheloe J Nelson JG Accuracy in self-reported health insurance coverage among Medicaid enrollees Inquiry 2008 45 4 438 456 19209838 10.5034/inquiryjrnl_45.04.438

[B25] Cameron AC Trivedi PK Microeconometrics: Methods and applications 2005 Cambridge, MA: Cambridge University Press

